# A detailed dosimetric comparative study of IMRT and VMAT in normal brain tissues for nasopharyngeal carcinoma patients treated with radiotherapy

**DOI:** 10.3389/fradi.2023.1190763

**Published:** 2023-05-18

**Authors:** Kainan Shao, Shuang Zheng, Yajuan Wang, Xue Bai, Hongying Luo, Fenglei Du

**Affiliations:** ^1^ Department of Radiation Physics, Zhejiang Cancer Hospital, Hangzhou, China; ^2^ School of Media and Design, Hangzhou Dianzi University, Hangzhou, China; ^3^ Faculty of Nuclear Science and Technology, University of South China, Hengyang, China

**Keywords:** nasopharyngeal carcinoma, radiotherapy, CT brain template, dosimetry, cognition

## Abstract

**Background:**

Radiotherapy (RT) is the primary treatment for nasopharyngeal carcinoma (NPC). However, it can cause implicit RT-induced injury by irradiating normal brain tissue. To date, there have been no detailed reports on the radiated exact location in the brain, the corresponding radiation dose, and their relationship.

**Methods:**

We analyzed 803 Chinese NPC patients treated with RT and used a CT brain template in a Montreal Neurological Institute (MNI) space to compare the group differences in RT dose distribution for different RT technologies (IMRT or VMAT).

**Results:**

Brain regions that received high doses (>50 Gy) of radiation were mainly located in parts of the temporal and limbic lobes, where radioactive damage often occurs. Brain regions that accepted higher doses with IMRT were mainly located near the anterior region of the nasopharyngeal tumor, while brain regions that accepted higher doses with VMAT were mainly located near the posterior region of the tumor. No significant difference was detected between IMRT and VMAT for T1 stage patients. For T2 stage patients, differences were widely distributed, with VMAT showing a significant dose advantage in protecting the normal brain tissue. For T3 stage patients, VMAT showed an advantage in the superior temporal gyrus and limbic lobe, while IMRT showed an advantage in the posterior cerebellum. For T4 stage patients, VMAT showed a disadvantage in protecting the normal brain tissue. These results indicate that IMRT and VMAT have their own advantages in sparing different organs at risk (OARs) in the brain for different T stages of NPC patients treated with RT.

**Conclusion:**

Our approach for analyzing dosimetric characteristics in a standard MNI space for Chinese NPC patients provides greater convenience in toxicity and dosimetry analysis with superior localization accuracy. Using this method, we found interesting differences from previous reports: VMAT showed a disadvantage in protecting the normal brain tissue for T4 stage NPC patients.

## 1. Introduction

Nasopharyngeal carcinoma (NPC) is one of the most common malignant tumors in the head and neck, which occurs in the nasopharyngeal epithelium. It has distinct geographical distribution characteristics and is particularly popular in southern China and Southeast Asia (1, 2). According to the International Agency for Research on Cancer (IARC), a number of 129,079 cases of NPC were newly diagnosed worldwide in 2018 (3), which increased to 133,354 in 2020 (https://gco.iarc.fr/today/online-analysis-table). NPC is a malignancy that has unique clinical biological profiles such as associated Epstein-Barr virus (EBV) infection and high radiosensitivity (2). Due to its high radiosensitivity and deep-seated anatomic location, definitive radiotherapy (RT) has long been recognized as the mainstream treatment modality for NPC since 1965 (4, 5).

The primary tumor in NPC had a special anatomical location and was surrounded by or overlapping with many organs at risk (OARs), making it difficult to achieve adequate tumor coverage while respecting the recommended dose constraints. Part of these OARs are unavoidably radiated, which can result in high doses being delivered, particularly in the case of advanced tumors (6, 7). Although the guiding principle of RT should always be As Low As Reasonably Practicable for OARs (8), normal tissue damages would occur in cases in which there is difficulty in achieving adequate tumor coverage while respecting the recommended dose constraints. This has been regarded as one of the most serious complications of RT in the past few decades and has received increasing attention with the increase in survival rate and favorable outcomes of NPC (6, 9–11). To date, a series of studies have explored the RT-induced structural and functional abnormalities in NPC patients, including changes in the volume of the whole-brain gray matter, bilateral temporal lobes, and ventricles (7, 12), hippocampal atrophy (13), and cognitive dysfunctions (9, 10, 14, 15).

In these reports, there was a notable focus on the general cognitive outcomes of NPC patients following RT, but little attention was given to the specific locations where structural and functional abnormalities occur, their associated cognitive dysfunctions, and the corresponding radiation exposure doses. The complexity of precisely correlating these three factors may have contributed to this gap in research.

Fortunately, the most widely used Colin27 and the Montreal Neurological Institute (MNI) 152 standard-space magnetic resonance imaging (MRI) T1-weighted structural template (shortened as MNI152 template) (16–21), which is often employed as the standardized coordinate system in neuroimaging studies, provided a very referential research direction and useful tool to more precisely explore the relationships between the structure, dosimetry parameters, and the cognitive function.

However, most studies that have analyzed MRI data to explore RT-related structural or functional abnormalities have had relatively small sample sizes. This is likely due to the fact that MRI, which is slower and more expensive than computed tomography (CT), is not a routine imaging modality for most NPC patients. CT, which is used to calculate the dose of radiotherapy in the Treatment Planning System (TPS) based on electron density, is the preferred imaging modality for NPC patients. However, the lack of sufficient soft tissue contrast on CT images makes it challenging to directly transform the images to the MNI152 template, which could result in a loss of information and representativeness if studies only included individuals with MRI whole-brain images and neglected those who only received CT scans. This presents a challenge for researchers studying the relationship between cognition and corresponding dosimetric characteristics.

Therefore, to investigate the direct relationship between dosimetric characteristics and cognitive dysfunction of specific brain structures radiated during RT for NPC patients, with a large sample size, a standard CT brain template is needed to substitute for the MNI152 template. The MNI standard-space CT brain template can provide a great convenience for analyzing RT-related CT and dosimetry data.

Brain templates created in the CT modality have limited reports in the literature, with only a few generated for specific cohorts of individuals. For example, Christopher Rorden et al. (22) developed an axial CT template for the cohort with ages similar to what is commonly seen in stroke, based on 35 healthy old individuals. A bimodal MR-CT head template was created for neonates (23). And recently, Deepthi Rajashekar et al. generated a high-resolution FLAIR atlas (from 136 healthy old subjects) and non-contrast CT atlas (from 47 patients with acute ischemic stroke) for the elderly (24). John Muschelli (25) created an unbiased CT template with 130 patients from the CQ500 dataset (26). These templates allow studies where only CT scans are available.

In this study, we utilized the CT brain template developed by Christopher Rorden et al. (22) in the MNI space. Spatial normalization of NPC patients' CT brain scans, and corresponding dose distribution maps were performed by using this specific CT brain template. This enabled us to accurately locate irradiated brain areas and reveal the dose distribution of these areas. Further, dosimetry characteristics of specific normal brain tissues for NPC patients treated with different irradiation methods were analyzed.

## 2. Materials and methods

This retrospective study was approved by the Ethics institutional review board of the Zhejiang Cancer Hospital. The data were anonymous, and the requirement for informed consent was therefore waived.

### 2.1. Participants

A total of 803 newly diagnosed histopathology-proven NPC patients who had CT scans covering the entire brain volume between December 2014 and November 2019 were retrospectively collected from our center.

All patients fulfilled the following inclusion criteria: not a recurrent NPC, without intracranial invasion, without brain tumors or metastases, without prior substantial head trauma, without current or past substance abuse or psychoactive drugs, without viral hepatitis, without neurological or psychiatric disorders, without positive human immunodeficiency virus status, without other severer systematic diseases, and without other malignant diseases that impacted prognosis of the patient.

The demographic characteristics of the 803 patients, including 567 males and 236 females, with a median age of 53 years, were shown in Table 1. The clinical T stages of NPC for the patients were classified according to the seventh edition of the International Union against Cancer/American Joint Committee on Cancer staging system, with one case classified as T0, 81 cases as T1 (median age of 55 years), 98 cases as T2 (median age of 51 years), 416 cases as T3 (median age of 52 years), and 207 cases as T4 (median age of 55 years).

**Table 1:** Demographic characteristics of 803 patients.

	T categories				
	T0	T1	T2	T3	T4
Number	1	81	98	416	207
**Gender**					
Male	—	61	61	294	151
Female	1	20	37	122	56
**Age (Y)**					
Median age	55	55	51	52	55
Range	—	28–77	26–77	18–80	25–84
**Radiotherapy**					
IMRT	—	57	66	245	129
VMAT	1	24	32	171	78
Nasopharynx RT dose (Gy)	70.4	70.5 (67.2–73.6)	70.6 (69–72.6)	70.4 (69–72.6)	70.4 (66.9–74.2)
**CT scanner**					
GE	—	48	50	211	110
Philips	1	33	48	205	97

### 2.2. CT data acquisition

All CT scans were acquired using a Philips Brilliance CT Big Bore or a GE LightSpeed RT CT scanner. Patients were positioned in the supine position and immobilized with a thermoplastic mask. High-resolution contrast-enhanced helical CT scans were then obtained with an x-ray tube voltage of 120 kV. The slice thickness was set as 3 mm or 5 mm in the Philips CT scanner and 2.5 mm or 5 mm in the GE CT scanner.

### 2.3. Radiotherapy

CT images acquired were transmitted to the Raystation 4.5 (RaySearch Laboratories, Sweden) Treatment Planning System (TPS) for target volume and OARs delineation. Planning target volumes (PTVs) for all patients were delineated slice-by-slice on contrast-enhanced CT images by radiation oncologists, according to ICRU 50 (27) and 62 reports (28). The gross tumor volume (GTV) covers the primary tumor in the nasopharynx (GTVnx) and the nodal target volume in the neck (GTVnd). The clinical target volume (CTV) covers the high-risk clinical target volume (CTV1), and the preventive clinical target volume (CTV2). The corresponding PTVs were generated by extending 3–5 mm around GTVs or CTVs in the TPS to account for positioning errors.

The prescribed radiation doses were 67–74 Gy in 30–33 fractions at 2.12–2.35 Gy/fraction to the PTV of the GTVnx, 60–74 Gy to the PTV of GTVnd, 60–64 Gy to the PTV of the CTV1, and 52–56 Gy to the PTV of the CTV2. The NPC radiation therapy was executed with a simultaneous integrated boost (SIB) plan. All patients were treated with 1 fraction daily over 5 days per week.

Patients were treated with Intensity-Modulated Radiation Therapy (IMRT) or Volumetric Modulated Arc Therapy (VMAT) technique on a Varian (Varian 23EX or Trilogy) linac or a Elekta (Elekta Synergy) linac. IMRT (497 patients, 61.89%) and VMAT (306 patients, 38.11%) plans were created in Raystation TPS. In the IMRT plans, 7 (154 patients, 19.18%) or 9 (343 patients, 42.71%) static coplanar fields of 6MV x-rays which were separated at 52° or 40° apart were adopted. In the VMAT plans, 2 (300 patients, 37.36%) or 4 (6 patients, 0.75%) rotating arcs of the same energy were set from 182° to 178° clockwise and from 178° to 182° counterclockwise. These 4 arcs VMAT plans and 9 field IMRT plans were performed for some patients with relatively bigger and more complex-shaped target volumes. The overall clinical goals of all targets were set as at least 95% of prescribed doses to planned targets.

The dose received by each OAR was restricted according to the ICRU83 Report (29) and RTOG0225 protocol (30). In clinical practice, our principle for plan designing was to ensure that the target area dose was sufficient while the doses to OAR were as low as possible. However, not all patients can meet such requirements, and there was often a trade-off in actual clinical practice to ensure that the target area dose or protection of OARs was prioritized. The treatment plans of all patients in this study and all patients treated at our center were reviewed and approved by physicians before treatment.

### 2.4. CT and RTdose images preprocessing

During radiotherapy, the dose distribution of the radiotherapy plan was calculated based on the CT electron density. Therefore, for the same patient, the dose distribution map corresponded completely to the CT image, that was, both were in the same coordinate system. Therefore, synchronization was also required in image registration and other image transformation processes.

At first, all patients' CT images and corresponding RT dose distribution images (RTdose images) were manually cropped to remove the excess signal from the neck and sides of the head by using 3D Slicer software (31) synchronously. Next, these CT and RTdose images were manually translated and rotated to align to the AC-PC line to roughly match the MNI template using the “Display” function in SPM8 (Statistical Parametric Mapping, SPM, http://www.fil.ion.ucl.ac.uk/spm/software/spm8/). This step was a totally manual registration, including only translation and rotation, thus it's a rigid registration. Thus, CT and RTdose images were in coarse alignment with each other.

### 2.5. Statistical analysis

Statistical analysis of descriptive characteristics of patients was done in SPSS (version 20). The normality of the age was verified by the Shapiro-Wilk test. The Wilcoxon test was executed for the comparison of the age parameter if it was non-normally distributed. Otherwise, the ANOVA test was adopted. The chi-square analysis was performed on the qualitative variables (gender, RT techniques) to assess the differences among different T stages of patients. *p* < 0.05 was considered statistically significant.

Patients' preprocessed CT images were first registered to the CT brain template created by Christopher Rorden et al. (22) with a resolution of 2 mm × 2 mm × 2 mm, and the same transformations were applied to the corresponding RTdose images. All the registrations were accomplished using SPM8. The registration algorithm employed by SPM typically starts with a linear affine registration step that aligns the source and target images globally by applying translations, rotations, and shearing. This is followed by an iterative nonlinear local warping transformation that aims to minimize the sum of squared differences between the source and target images (32). Thus, it's a deformable registration.

According to the study by Chen X et al., the permutation test with Threshold-Free Cluster Enhancement (TFCE) achieves the best balance between family-wise error rate (under 5%) and test-retest reliability/replicability, thus outperforming other multiple comparison correction strategies (33). Thus, the analysis of dosimetric characteristics of different RTtechs in the brain for all patients and each T stage of patients was explored in two-sample *t*-tests within the dpabi toolbox (34) (DPABI, Version 5.1), by using a permutation TFCE test (number of permutations = 5,000, thresholded at FWE *p* < 0.05) with age and gender as covariates.

Brain regions of differences were executed by xjView toolbox (http://www.alivelearn.net/xjview).

## 3. Results

### 3.1. Registration of patients' Ct and RTdose images

**Figure 1:**
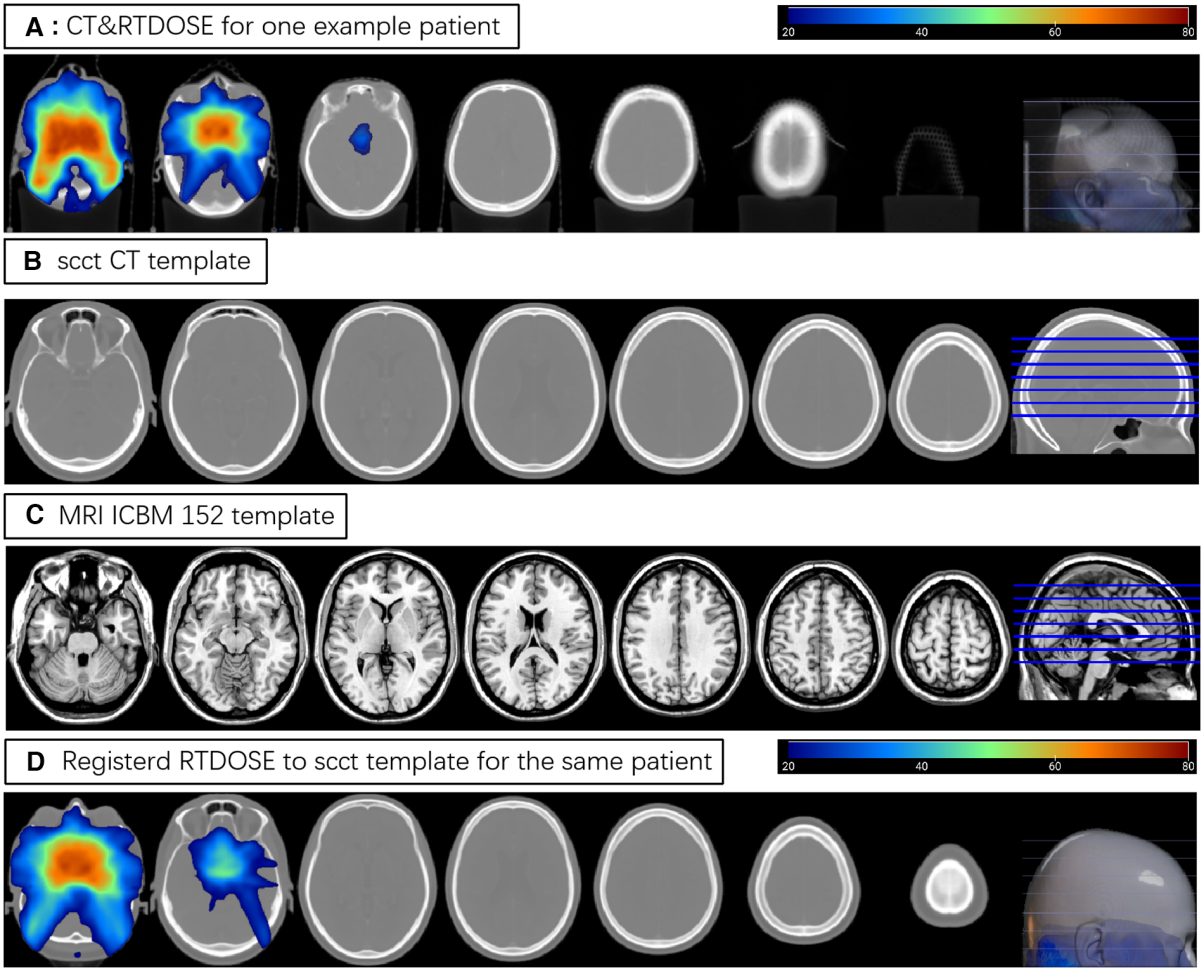
(**A**) shows some slices of the CT and RTdose images for one example patient; (**B**) shows slices of the CT template created by Christopher Rorden; (**C**) shows slices of the MRI T1 template in MNI space created by ICBM; (**D**) shows slices of the RTdose images registered to the CT template created by Christopher Rorden for the same patient.

The registration of patients' CT and RTdose images to the CT brain template created by Christopher Rorden et al. (22) in MNI space was illustrated in Figure 1. The upper row (A) shows some slices of the CT and RTdose images for one example patient in the original individual space; (B) shows slices of the CT template created by Christopher Rorden et al. (22); (C) shows slices of the MRI T1 template in MNI space created by ICBM; (D) shows slices of the RTdose images registered to the CT template created by Christopher Rorden for the same patient.

### 3.2. Demographic characteristics

The Shapiro-Wilk test was applied to ages of patients in different T stages. Results showed *ps* > 0.05 for all T1–T4 stages (*p* = 0.333, 0.896, 0.214, and 0.403 for T1–T4 stage, respectively), indicating that the variable of age was normally distributed among T1–T4 stage patients. One-way ANOVA was adopted to analyze the difference in ages of all patients, and the result showed a significant difference among T stages (F (3,801) = 2.812, *p* = 0.038). Bonferroni's *post hoc* test revealed that only the difference between ages of T3 and T4 stages was significant (*p* = 0.045). When the Shapiro-Wilk test was applied to the ages of all patients, the result (*p* = 0.015) indicated that the variable of age was non-normally distributed. Thus, the Kruskal-Wallis nonparametric test was applied to ages of all patients. A similar result was found. The difference among the four T stages was significant (*p* = 0.041). Further nonparametric analysis revealed that only the difference between ages of T3 and T4 stage was significant (*p* = 0.01). The chi-square analysis performed on the qualitative variables (gender, RT techniques) showed that the differences in the gender and RT technique among the 4 T stages of patients were not significant (*p = *0.194 for ages and *p = *0.151 for RT techniques).

Although some of the variables of age, gender, and RT techniques were not significantly different among stages, they were still used as covariates in the group-wise analysis of dosimetry characteristics.

### 3.3. Average of RTdose images

**Figure 2:**
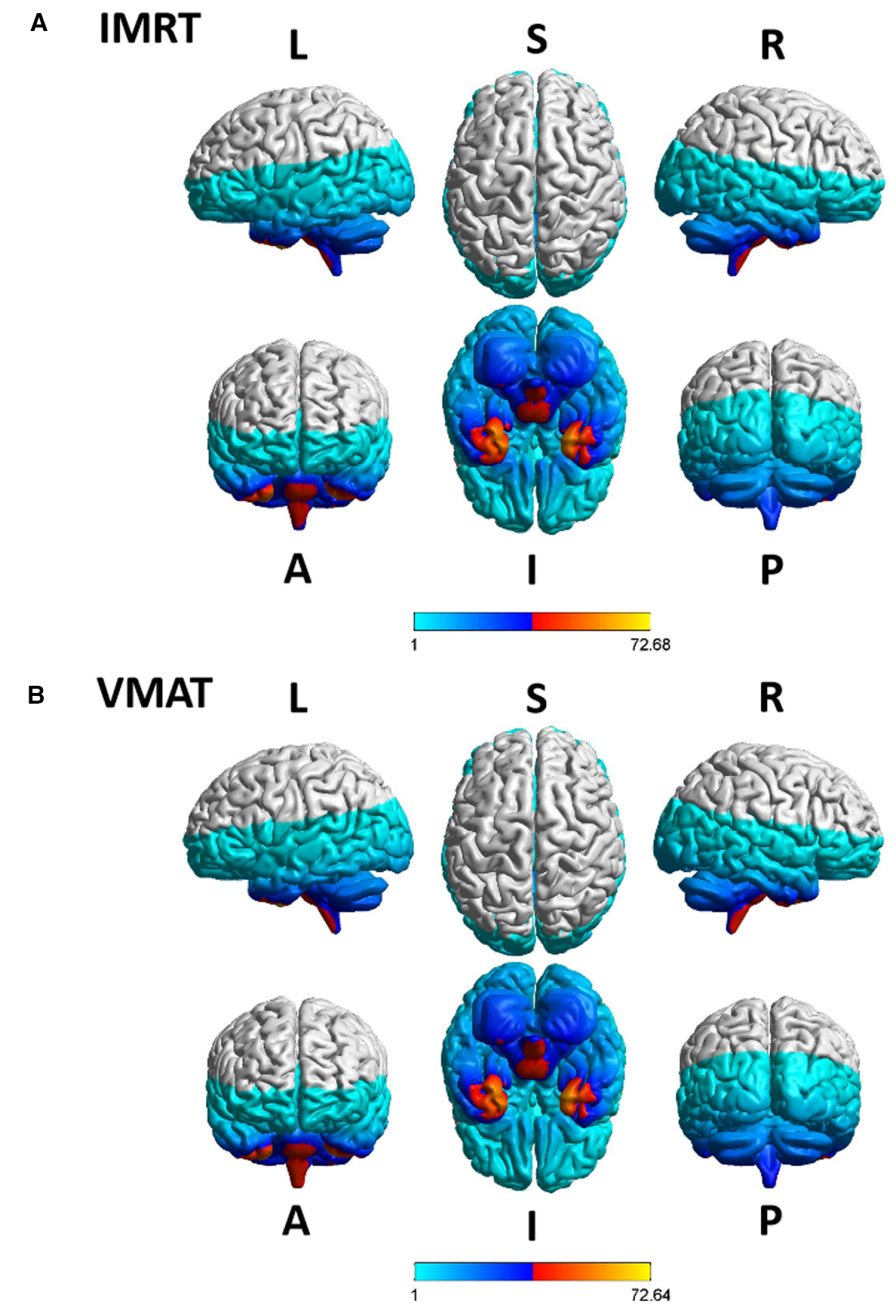
The preview of the projection of average RTdose maps into the ICBM 152 brain surface template for each RTtech of patients. (**A**) shows the preview of patients treated by IMRT (including 497 patients); (**B**) shows the preview of patients treated by VMAT (including 306 patients). The dose display range was thresholded as 1.0 Gy–80 Gy. (L, left; R, right; S, superior; I, inferior; A, anterior; P, posterior).

The overview of RTdose maps was derived by averaging patients' RTdose images for each RTtech of patients. The RTdose maps were projected into the Colin27 MRI brain surface template in BrainNet viewer toolbox (35), and the schematic diagram was shown in Figure 2. (A) shows the preview of the IMRT RTtech; (B) shows the preview of VMAT RTtech. The dose display range was set as 1.0 Gy–80 Gy to make the demonstration of the difference clearer.

**Figure 3:**
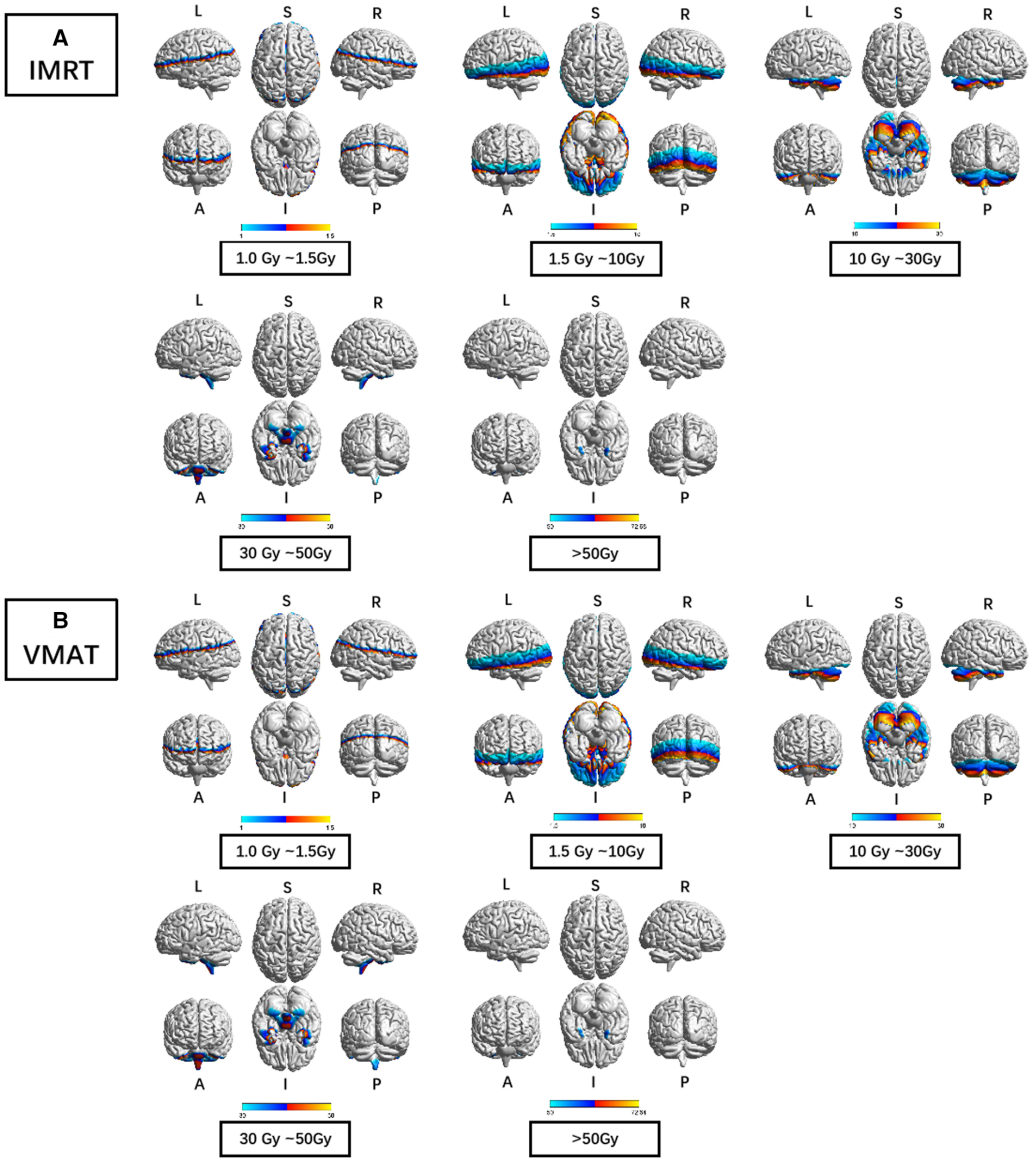
The preview of the projection of average RTdose maps into ICBM 152 brain surface template for each RTtech of patients. (**A**) shows the preview of patients treated by IMRT (including 497 patients); (**B**) shows the preview of patients treated by VMAT (including 306 patients). The dose display range was thresholded as 1.0–1.5 Gy, 1.5–10 Gy, 10–30 Gy, 30–50 Gy, and >50 Gy. (L: left; R: right; S: superior; I: inferior; A: anterior; P: posterior).

Furthermore, the dose display range was segregated into 5 subranges: 1.0–1.5 Gy, 1.5–10 Gy, 10–30 Gy, 30–50 Gy, and >50 Gy. The detailed description of brain regions covered by these subranges for each RTtech of patients was executed using xjView toolbox (http://www.alivelearn.net/xjview) separately, and the schematic diagram was shown in Figure 3.

Brain regions that covered by the 1.0–1.5 Gy dose distribution map of patients treated by the IMRT RTtech included part of the frontal lobe (part of the sub gyral, the precentral gyrus, the inferior and medial frontal gyrus, the middle frontal gyrus, the paracentral lobule, and the superior frontal gyrus), the sub-lobar (part of the extra nuclear, the insula, the corpus callosum, the lateral ventricle, a bit of the caudate, the claustrum, the lentiform nucleus and the thalamus), the parietal lobe (part of the sub gyral, the precuneus, the postcentral gyrus, the inferior parietal lobule, the supramarginal gyrus), the temporal lobe (part of the superior temporal gyrus, the middle temporal gyrus and the transverse temporal gyrus), the limbic lobe (part of the cingulate gyrus and the anterior cingulate, a bit of the posterior cingulate), the cuneus, and the occipital lobe (part of the superior occipital gyrus).

Brain regions that covered by the 1.5–10 Gy dose distribution map of patients treated by the IMRT RTtech included part of the temporal lobe (part of the sub gyral, the middle temporal gyrus, the superior temporal gyrus, the inferior temporal gyrus, and a bit of the transverse temporal gyrus), the occipital lobe (part of the middle occipital gyrus, the cuneus, the lingual gyrus, the fusiform, the inferior occipital gyrus, a bit of the precuneus and the superior occipital gyrus), the sub-lobar (part of the extra nuclear, the lentiform nucleus, the insula, the thalamus, the lateral ventricle, the corpus callosum, the caudate, the claustrum and the third ventricle), the frontal lobe (part of the inferior frontal gyrus, the middle frontal gyrus, the superior frontal gyrus, the medial frontal gyrus, the subcallosal gyrus, the orbital gyrus, the rectal gyrus and a bit of the precentral gyrus), the limbic lobe (the parahippocampal gyrus, part of the posterior cingulate and the anterior cingulate), the brainstem (part of the midbrain and the red nucleus), a bit of the cerebellum anterior lobe (the culmen), and the cerebellum posterior lobe, a bit of the parietal lobe and the frontal-temporal space.

Brain regions that covered by the 10–30 Gy dose distribution map of patients treated by the IMRT RTtech included part of the cerebellum anterior lobe (part of the declive, the culmen, the whole dentate, the whole nodule, the whole fastigium, and part of the cerebellar lingual), the temporal lobe (part of the fusiform gyrus, the inferior temporal gyrus, the superior temporal gyrus and the middle temporal gyrus), the cerebellum posterior lobe (part of the declive, the cerebellar tonsil, the whole tuber, the whole inferior semi-lunar lobule, the pyramis, the uvula, the declive of vermis, the uvula of vermis, the pyramis of vermis and the tuber of vermis), the occipital lobe (part of the fusiform gyrus, lingual gyrus, and the inferior occipital gyrus), the limbic lobe (part of the parahippocampal gyrus and the uncus), the frontal lobe (part of the sub gyral, the rectal gyrus, the inferior frontal gyrus, the orbital gyrus, and the medial frontal gyrus), the brainstem (part of the pons, the midbrain, and the medulla), a bit of the sub lobar (the whole fourth ventricle and the lateral ventricle).

Brain regions that covered by the 30–50 Gy dose distribution map of patients treated by the IMRT RTtech included part of the cerebellum posterior lobe (part of the cerebellar tonsil), the temporal lobe (part of the superior temporal gyrus, the middle temporal gyrus, the inferior temporal gyrus, and a bit of the sub gyral), the limbic lobe (part of the uncus and the parahippocampal gyrus), and the brainstem (part of the pons and the medulla).

Brain regions that covered by the >50 Gy dose distribution map of patients treated by the IMRT RTtech included part of the temporal lobe (part of the superior temporal gyrus and a bit of the inferior and middle temporal gyrus) and the limbic lobe (part of the uncus).

Brain regions that covered by the 1.0–1.5 Gy dose distribution map of patients treated by the VMAT RTtech included part of the frontal lobe (part of the sub gyral, the inferior and medial frontal gyrus, the precentral gyrus, the middle frontal gyrus and the superior frontal gyrus), the sub-lobar (part of the extra nuclear, the insula, the corpus callosum, the lateral ventricle, a bit of the caudate and the claustrum), the parietal lobe (part of the sub gyral, the precuneus, the postcentral gyrus, the inferior parietal lobule, the supramarginal gyrus, the angular gyrus), the limbic lobe (part of the cingulate gyrus and the anterior cingulate, a bit of the posterior cingulate), the temporal lobe (part of the superior temporal gyrus, the middle temporal gyrus and the transverse temporal gyrus), the occipital lobe (part of the superior occipital gyrus and the cuneus).

Brain regions that covered by the 1.5–10 Gy dose distribution map of patients treated by the VMAT RTtech included part of the temporal lobe (part of the sub gyral, the middle temporal gyrus, the superior temporal gyrus, the inferior temporal gyrus, and a bit of the transverse temporal gyrus), the sub-lobar (part of the extra nuclear, the insula, the lentiform nucleus, the thalamus, the lateral ventricle, the corpus callosum, the caudate, the claustrum and the third ventricle), the occipital lobe (part of the middle occipital gyrus, the cuneus, the lingual gyrus, the fusiform, the inferior occipital gyrus, a bit of the precuneus and the superior occipital gyrus), the frontal lobe (part of the inferior frontal gyrus, the middle frontal gyrus, the superior frontal gyrus, the medial frontal gyrus, the subcallosal gyrus, the orbital gyrus, the rectal gyrus and a bit of the precentral gyrus), the limbic lobe (the parahippocampal gyrus, part of the posterior cingulate and the anterior cingulate), the brainstem (part of the midbrain and the red nucleus), a bit of the cerebellum anterior lobe (the culmen, and the culmen of vermis), and a bit of the parietal lobe and the frontal-temporal space.

Brain regions that covered by the 10–30 Gy dose distribution map of patients treated by the VMAT RTtech included part of the cerebellum anterior lobe (part of the declive, the culmen, the whole dentate, the whole nodule, part of the cerebellar lingual and the whole fastigium), the temporal lobe (part of the fusiform gyrus, the inferior temporal gyrus, the superior temporal gyrus and the middle temporal gyrus), the cerebellum posterior lobe (part of the declive, the inferior semi-lunar lobule, the cerebellar tonsil, the whole tuber, the pyramis, the uvula, the declive of vermis, the uvula of vermis, the pyramis of vermis and the tuber of vermis), the occipital lobe (part of the fusiform gyrus, lingual gyrus, the inferior occipital gyrus, the middle occipital gyrus), the limbic lobe (part of the parahippocampal gyrus and the uncus),the brainstem (part of the pons, the midbrain, and the medulla), the frontal lobe (part of the sub gyral, the rectal gyrus, the orbital gyrus and the inferior frontal gyrus), a bit of the sub lobar (the whole fourth ventricle and the lateral ventricle).

Brain regions that covered by the 30–50 Gy dose distribution map of patients treated by the VMAT RTtech included part of the cerebellum posterior lobe (part of the cerebellar tonsil and a bit of the inferior semi-lunar lobule), the temporal lobe (part of the superior temporal gyrus, the middle temporal gyrus, the inferior temporal gyrus, and a bit of the sub gyral), the limbic lobe (part of the uncus and the parahippocampal gyrus), and the brainstem (part of the pons and the medulla).

Brain regions that covered by the >50 Gy dose distribution map of patients treated by the VMAT RTtech included part of the temporal lobe (part of the superior temporal gyrus and a bit of the inferior and middle temporal gyrus) and the limbic lobe (part of the uncus).

To clarify the volumes covered by VMAT and IMRT separately in different dose sub-ranges, we summarized the voxel sizes of each dose sub-range for VMAT and IMRT, which were demonstrated in Table 2.

**Table 2:** Volumes covered by VMAT and IMRT separately in different dose sub-ranges in the brain. voxel size: 2 mm * 2 mm * 2 mm.

RTtech	Voxels in different dose ranges (Gy)				
	1.0–1.5 Gy	1.5–10 Gy	10–30 Gy	30–50 Gy	>50 Gy
IMRT	33,701	79,386	38,617	9,534	3,480
VMAT	31,488	83,032	37,643	9,649	3,414

We generated a brain mask by excluding voxels outside the ICBM brain template, with each voxel size of 2 mm * 2 mm * 2 mm. Using this mask, we calculated the voxel sizes of the dose distribution in different dose sub-ranges within the brain. For IMRT, we observed 33,701, 79,386, 38,617, 9,534, and 3,480 voxels in the 1.0 Gy–1.5 Gy, 1.5 Gy–10 Gy, 10 Gy–30 Gy, 30 Gy–50 Gy, and >50 Gy sub-ranges, respectively. For VMAT, we obtained 31,488, 83,032, 37,643, 9,649, and 3,414 voxels in the same sub-ranges, respectively. We noted that the dose distribution in the 1.5 Gy–10 Gy range was consistent with the commonly held view that VMAT covers a larger low dose volume than IMRT.

### 3.4. Differences in RTdose images between different RTtechs

**Figure 4:**
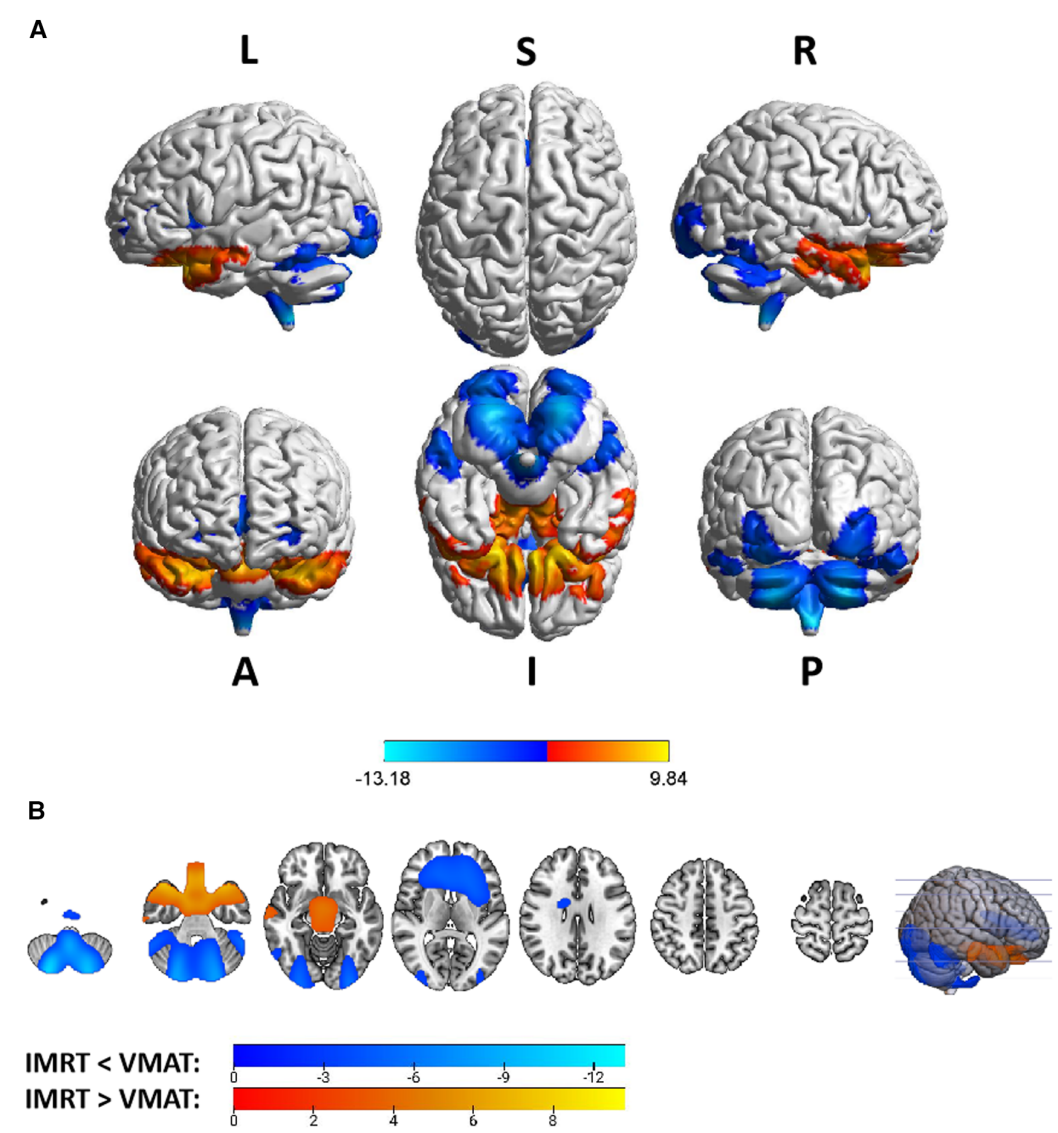
The preview of RTdose differences between patients treated by IMRT vs. VMAT that explored in a two-sample t-test by using a permutation TFCE test (FWE *p *< 0.05). (**A**) shows the projection of the two-sample t-test result of all patients (IMRT vs. VMAT) into the Colin27 MRI brain surface template, and (**B**) shows some slices of the difference distributed in the axial direction. (L, left; R, right; S, superior; I, inferior; A, anterior; P, posterior).

Group differences in RTdose data for different RTtechs of all patients were shown in Figure 4. The detailed brain regions of the group differences were executed by xjView toolbox (http://www.alivelearn.net/xjview). Brain regions in which the delivered dose was significantly higher in patients treated with the IMRT method than that with VMAT were located in part of the temporal lobe (part of the superior temporal gyrus, the middle temporal gyrus, a little part of the sub gyral, the inferior temporal gyrus, and a bit of the fusiform gyrus), the frontal lobe (part of the inferior frontal gyrus, most of the rectal gyrus, and the medial frontal gyrus, a little part of the sub gyral, the subcallosal gyrus, the orbital gyrus and the middle frontal gyrus), the limbic lobe (part of the parahippocampal gyrus and the uncus), the brainstem (part of the midbrain and a bit of the pons and the substania nigra), and a little part of the sub lobar (a bit of the extra nuclear, the lateral ventricle).

Brain regions in which the delivered dose was significantly lower in patients treated with the IMRT method than that with VMAT were located in part of the cerebellum posterior lobe (part of the declive, the inferior semi-lunar lobule, the cerebellar tonsil, the pyramis, the uvula, and a bit of the tuber, the declive of vermis, the uvula of vermis and the pyramis of vermis), the occipital lobe (part of the middle occipital gyrus, the fusiform gyrus, the lingual gyrus, the inferior occipital gyrus, and a bit of the cuneus), the cerebellum anterior lobe (part of the culmen, most of the dentate, the nodule and the fastigium), the temporal lobe (part of the fusiform gyrus, a bit of the sub gyral and the inferior temporal gyrus), the brainstem (part of the medulla and the pons), the frontal lobe (part of the of the sub gyral, the medial frontal gyrus, the inferior and superior frontal gyrus, and the precentral gyrus), the sub lobar (most of the fourth ventricle, part of the extra-nuclear, the insula, the lateral ventricle, the caudate, the Lentiform nucleus and the claustrum), and the limic lobe (part of the anterior cingulate).

Voxel sizes, the peak value and the location of the peak value in MNI space for each brain regions described above were depicted in Table 3.

**Table 3:** Brain regions showed significant differences in RTdose images between RTtechs (IMRT vs. VMAT) in all patients in a two-sample t-test by using a permutation threshold-free cluster enhancement test (number of permutations = 5,000, FWE *p* < 0.05), voxel size: 2*2*2.

Regions	Voxels	Peak T value	MNI coordinate		
			*x*	*y*	*z*
**IMRT < VMAT**					
Cerebellum Posterior Lobe	9,153	−11.72	26	−86	−48
Frontal Lobe	4,264	−5.31	14	38	2
Occipital Lobe	4,222	−7.06	24	−94	−26
Sub-lobar	3,859	−5.79	−2	−50	−44
Cerebellum Anterior Lobe	2,067	−6.81	−18	−66	−34
Limbic Lobe	1,210	−5.27	10	38	2
Temporal Lobe	993	−5.68	−58	−54	−24
Medulla	337	−8.06	2	−48	−52
Pons	297	−5.29	0	−44	−42
**IMRT > VMAT**					
Temporal Lobe	4,592	8.30	−22	10	−26
Frontal Lobe	3,212	9.27	−12	12	−26
Limbic Lobe	1,771	9.12	14	2	−24
Midbrain	1,061	8.07	−4	−10	−22
Pons	317	8.14	6	−10	−24
Sub-lobar	203	5.24	26	−6	−24

No significant difference was detected in RTdose data for different RTtechs of T1 patients.

**Figure 5:**
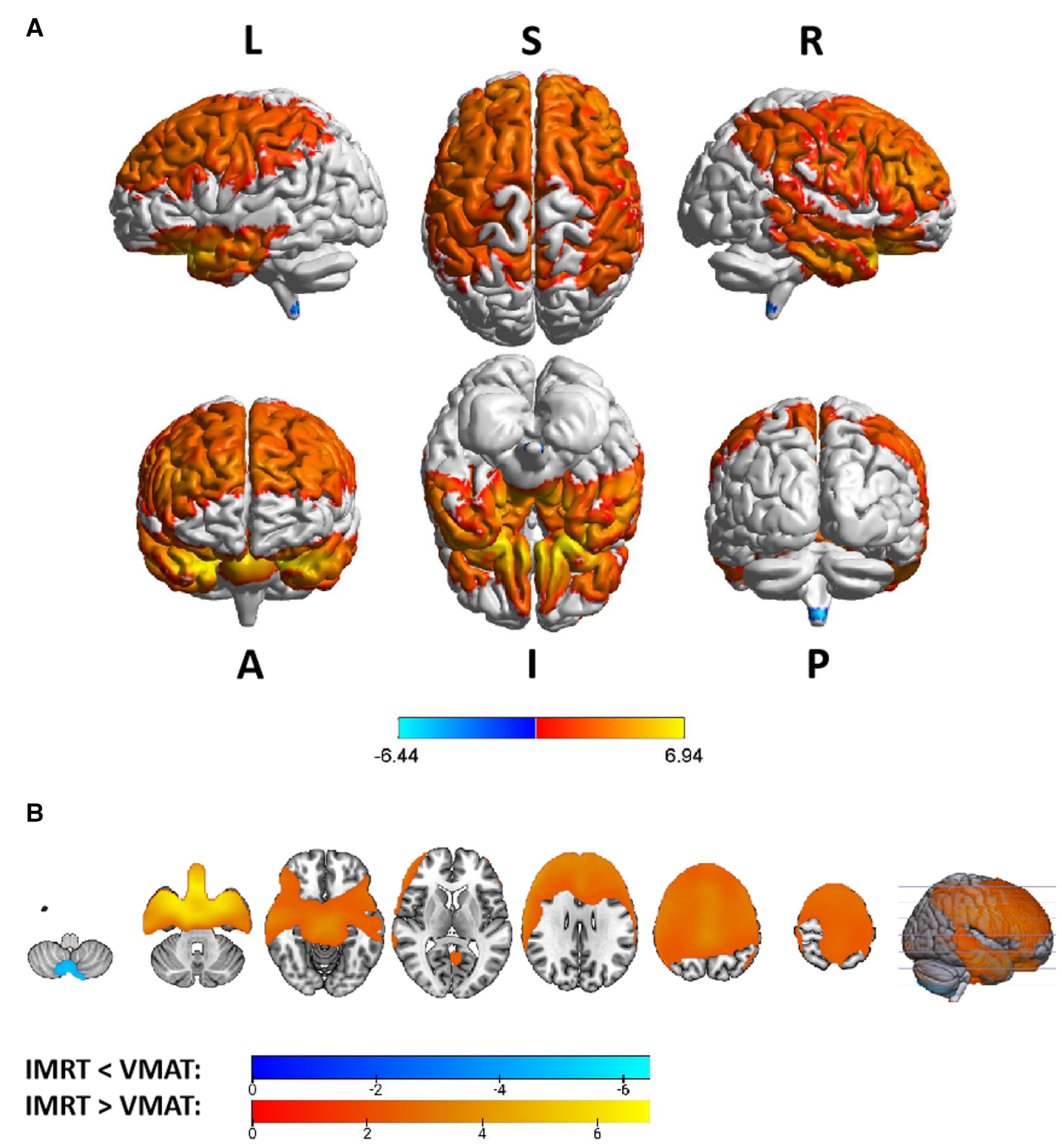
The preview of RTdose differences between patients treated by IMRT vs. VMAT that explored in a two-sample *t*-test by using a permutation TFCE test (FWE *p* < 0.05). (**A**) shows the projection of the two-sample t-test result for the T2 stage of patients (IMRT vs. VMAT) into the Colin27 MRI brain surface template, and (**B**) shows some slices of the difference distributed in the axial direction. (L, left; R, right; S, superior; I, inferior; A, anterior; P, posterior).

Group differences in RTdose data for different RTtechs of patients classified as T2 stage were shown in Figure 5. The detailed brain regions of the group differences were executed by xjView toolbox (http://www.alivelearn.net/xjview). Brain regions in which the delivered dose was significantly higher in patients treated with the IMRT method than that with VMAT were located in a part of the frontal lobe (part of the middle frontal gyrus, the superior frontal gyrus, the precentral gyrus, the medial frontal gyrus, the inferior frontal gyrus, the paracentral lobule, most of the rectal gyrus, the subcallosal gyrus, and the orbital gyrus), the temporal lobe (part of the sub gyral, the middle temporal gyrus, the superior temporal gyrus, the inferior temporal gyrus, and the fusiform gyrus), the parietal lobe (part of the postcentral gyrus, the inferior parietal lobule, the precuneus, the superior parietal lobule, and a bit of the supramarginal gyrus), the limbic lobe (part of the cingulate gyrus, parahippocampal gyrus, the uncus, and a bit of the anterior cingulate and the posterior cingulate), the brainstem (part of the midbrain and the pons), a bit of the sub lobar (a bit of the extra nuclear, the lateral ventricle, and the lentiform nucleus), and a bit of the cerebellum anterior lobe (a bit of the culmen), and a little bit of the occipital lobe (a bit of the lingual gyrus).

Brain regions in which the delivered dose was significantly lower in patients treated with the IMRT method than that with VMAT were located in a part of the cerebellum posterior lobe (part of the inferior semi-lunar lobule).

Voxel sizes, the peak value and the location of the peak value in MNI space for each brain regions described above were depicted in Table 4.

**Table 4:** Brain regions showed significant differences in RTdose images between RTtechs (IMRT vs. VMAT) in T2 patients in a two-sample t-test by using a permutation threshold-free cluster enhancement test (number of permutations = 5,000, FWE *p* < 0.05), voxel size: 2*2*2.

Regions	Voxels	Peak T value	MNI coordinate		
			*x*	*y*	*z*
**IMRT < VMAT**					
Cerebellum Posterior Lobe	147	−5.46	6	−66	−54
**IMRT > VMAT**					
Frontal Lobe	41,049	6.92	−6	22	−30
Temporal Lobe	10,188	6.90	24	14	−28
Parietal Lobe	9,992	4.38	68	−18	36
Limbic Lobe	8,908	6.75	22	12	−30
Midbrain	1,846	5.95	−4	−10	−22
Sub-lobar	794	5.87	−22	−6	−26
Pons	785	6.12	2	−12	−26
Cerebellum Anterior Lobe	610	3.54	−14	−28	−18
Occipital Lobe	52	2.61	−14	−42	−4

**Figure 6:**
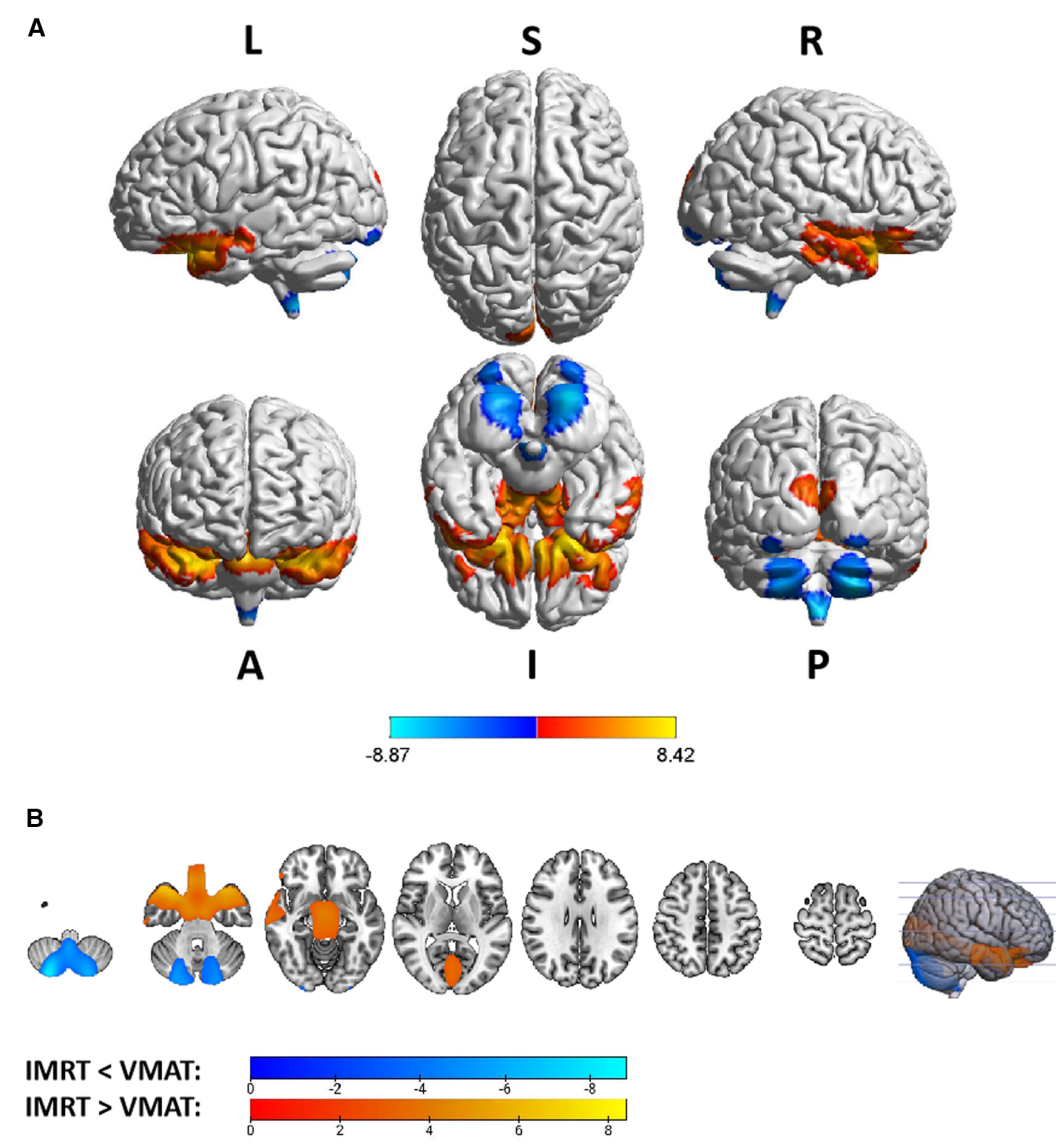
The preview of RTdose differences between patients treated by IMRT vs. VMAT that explored in a two-sample t-test by using a permutation TFCE test (FWE *p* < 0.05). (**A**) shows the projection of the two-sample t-test result for the T3 stage of patients (IMRT vs. VMAT) into the Colin27 MRI brain surface template, and (**B**) shows some slices of the difference distributed in the axial direction. (L, left; R, right; S, superior; I, inferior; A, anterior; P, posterior).

Group differences in RTdose data for different RTtechs of patients classified as T3 stage were shown in Figure 6. The detailed brain regions of the group differences were executed by xjView toolbox (http://www.alivelearn.net/xjview). Brain regions in which the delivered dose was significantly higher in patients treated with the IMRT method than that with VMAT were located in a part of the temporal lobe (part of the superior temporal gyrus, the middle temporal gyrus, a little part of the sub gyral and the inferior temporal gyrus, and a bit of the fusiform gyrus), the frontal lobe (part of the inferior frontal gyrus, the rectal gyrus, the subcallosal gyrus, a little part of the medial frontal gyrus, part of the orbital gyrus, and a bit of the middle frontal gyrus), the limbic lobe (part of the parahippocampal gyrus, the uncus, and the posterior cingulate), the occipital lobe (part of the cuneus and the lingual gyrus), the brainstem (part of the midbrain and the pons), the sub lobar (a bit of the lateral ventricle and the extra nuclear), a bit of the cerebellum anterior lobe (a bit of the culmen).

Brain regions in which the delivered dose was significantly lower in patients treated with the IMRT method than that with VMAT were located in a part of cerebellum posterior lobe (part of the inferior semi-lunar lobule, the pyramis, the uvula, the declive, the cerebellar tonsil, and the tuber), the occipital lobe (part of the the fusiform gyrus, the inferior occipital gyrus and the lingual gyrus), the cerebellum anterior lobe (part of the dentate), and the brainstem (part of the medulla).

Voxel sizes, the peak value and the location of the peak value in MNI space for each brain regions described above were depicted in Table 5.

**Table 5:** Brain regions showed significant differences in RTdose images between RTtechs (IMRT vs. VMAT) in T3 patients in a two-sample *t*-test by using a permutation threshold-free cluster enhancement test (number of permutations = 5,000, FWE *p* < 0.05), voxel size: 2*2*2.

Regions	Voxels	Peak T value	MNI coordinate		
			*x*	*y*	*z*
**IMRT < VMAT**					
Cerebellum Posterior Lobe	4,429	−8.87	30	−86	−48
Occipital Lobe	551	−4.62	24	−94	−26
Cerebellum Anterior Lobe	83	−4.24	22	−66	−34
Medulla	53	−5.27	2	−48	−52
**IMRT > VMAT**					
Temporal Lobe	5,059	7.67	30	18	−26
Frontal Lobe	2,941	7.66	16	12	−26
Limbic Lobe	2,012	8.01	−14	2	−24
Midbrain	1,317	7.39	4	10	−22
Occipital Lobe	1,201	3.82	0	−94	18
Pons	412	7.39	−6	−10	−24
Cerebellum Anterior Lobe	336	3.68	12	−30	−18
Sub-lobar	197	6.22	−22	−6	−24

**Figure 7:**
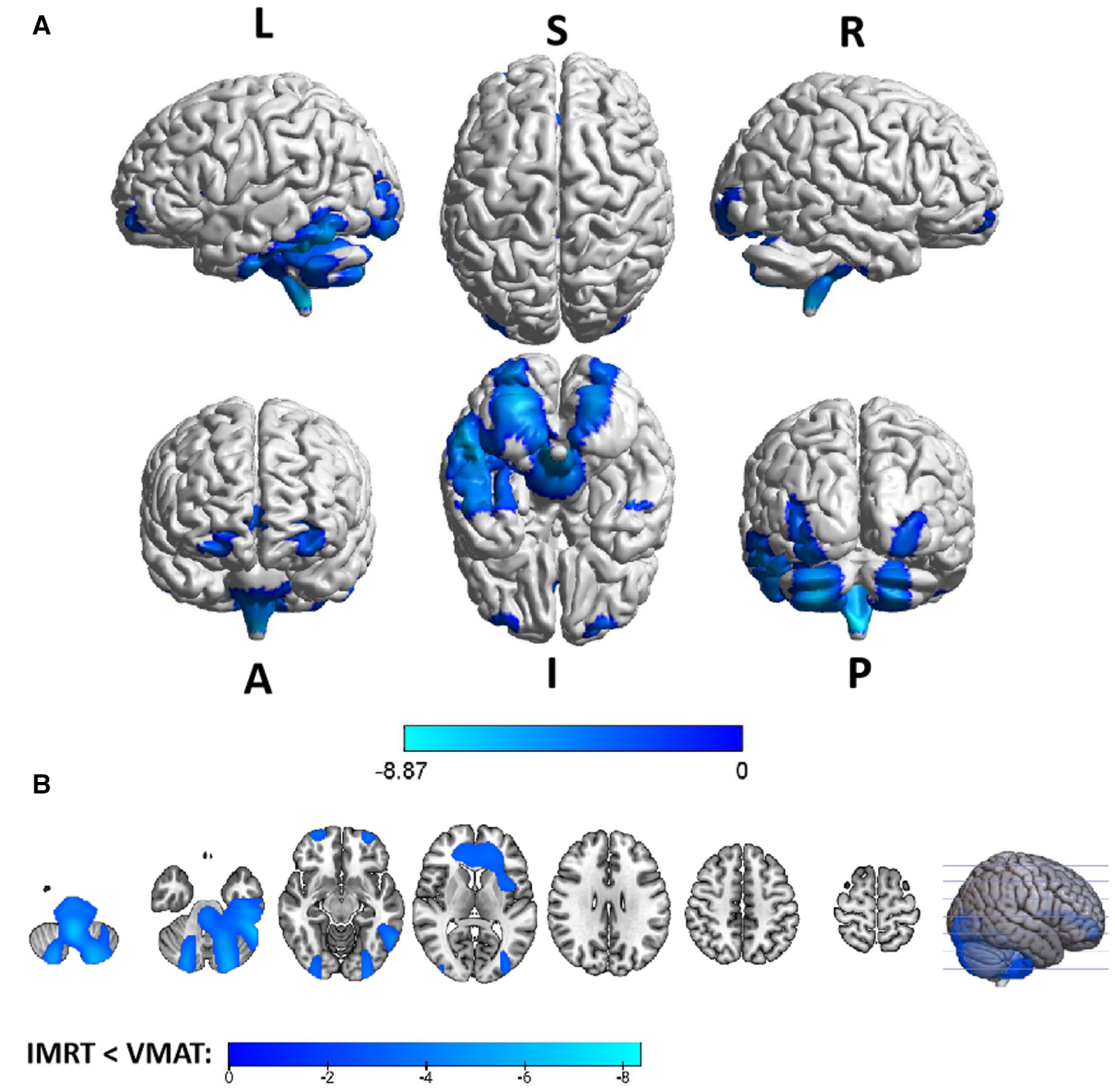
The preview of RTdose differences between patients treated by IMRT vs. VMAT that explored in a two-sample t-test by using a permutation TFCE test (FWE *p* < 0.05). (**A**) shows the projection of the two-sample *t*-test result for the T4 stage of patients (IMRT vs. VMAT) into the Colin27 MRI brain surface template, and (**B**) shows some slices of the difference distributed in the axial direction. (L, left; R, right; S, superior; I, inferior; A, anterior; P, posterior).

Group differences in RTdose data for different RTtechs of patients classified as T4 stage were shown in Figure 7. The detailed brain regions of the group differences were executed by xjView toolbox (http://www.alivelearn.net/xjview). No brain regions in which the delivered dose was significantly higher in patients treated with the IMRT method than that with VMAT were found.

Brain regions in which the delivered dose was significantly lower in patients treated with the IMRT method than that with VMAT were located in a part of the cerebellum posterior lobe (part of the cerebellar tonsil, the declive, the inferior semi-lunar lobule, the pyramis, the uvula, and the tuber), the occipital lobe (part the fusiform gyrus, the middle occipital gyrus, the inferior occipital gyrus, the lingual gyrus, and a bit of the cuneus), the cerebellum anterior lobe (part of the culmen, the dentate, and the nodule), the temporal lobe (a little part of the inferior temporal gyrus, the sub gyral, and the middle temporal gyrus), the frontal lobe (part of the sub gyral, the superior frontal gyrus, the medial frontal gyrus, the middle frontal gyrus, and a bit of the precentral gyrus), the brainstem (part of the pons and the medulla), the limbic lobe (part of the anterior cingulate, a little part of the parahippocampal gyrus, and a bit of the uncus), and the sub lobar (part of the fourth ventricle, part of the extra nuclear, the insula, the corpus callosum, the claustrum, and a bit of the lateral ventricle).

Voxel sizes, the peak value and the location of the peak value in MNI space for each brain regions described above were depicted in Table 6.

**Table 6:** Brain regions showed significant differences in RTdose images between RTtechs (IMRT vs. VMAT) in T4 patients in a two-sample *t*-test by using a permutation threshold-free cluster enhancement test (number of permutations = 5,000, FWE *p* < 0.05), voxel size: 2*2*2.

Regions	Voxels	Peak T value	MNI coordinate		
			*x*	*y*	*z*
**IMRT < VMAT**					
Cerebellum Posterior Lobe	7,381	−7.21	−24	−88	−46
Occipital Lobe	2,594	−5.39	−26	−92	−26
Frontal Lobe	2,399	−3.83	30	62	−16
Cerebellum Anterior Lobe	1,969	−5.88	−48	−44	−30
Temporal Lobe	1,940	−6.64	−58	−54	−24
Pons	1,060	−6.13	2	−32	−40
Limbic Lobe	981	−4.27	−38	−16	−36
Sub-lobar	719	−4.96	−2	−50	−44
Medulla	452	−6.41	0	−46	−52
**IMRT > VMAT**					
None					

### 3.5. ROI analysis

**Figure 8:**
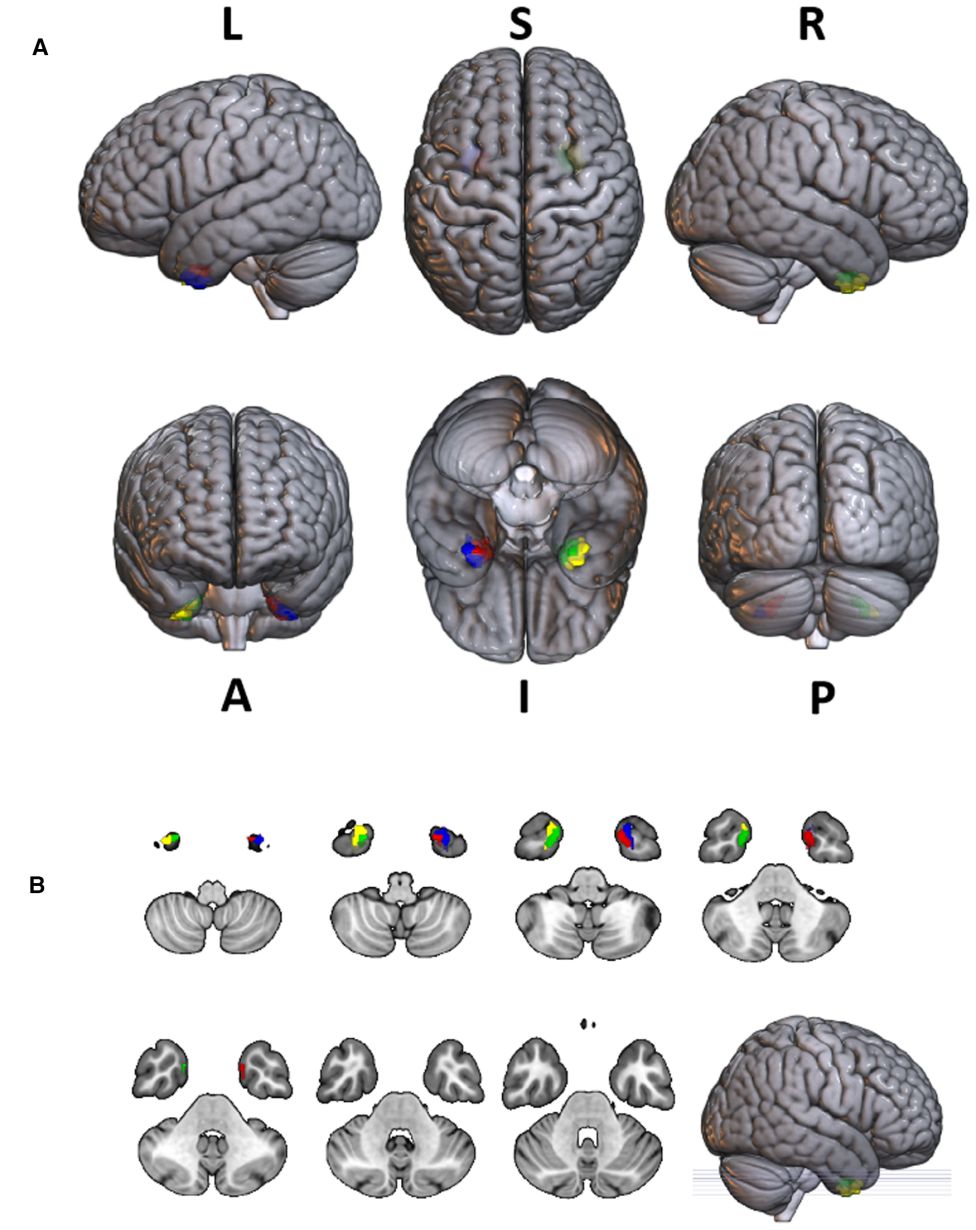
The projection of 4 ROIs (regions of interest) which received radiation doses >50 Gy to the SPM 152 MRI brain template. The radiation dose data were derived from the average of all patients. The ROI inked in red was part of the left limbic lobe that received radiation doses >50 Gy; The ROI inked in green was part of the right limbic lobe that received radiation doses >50 Gy; The ROI inked in blue was part of the left temporal lobe that received radiation doses >50 Gy; The ROI inked in yellow was part of the right temporal lobe that received radiation doses >50 Gy. (L, left; R, right; S, superior; I, inferior; A, anterior; P, posterior).

Four brain regions that received more than 50 Gy of radiation dose—the left temporal lobe, the right temporal lobe, the left limbic lobe, and the right limbic lobe—were selected as regions of interest (ROI), as shown in Figure 8. The mean radiation doses of these four ROIs were extracted for all patients, and a 2-sample t-test was conducted to investigate any differences in radiation dose between the two RT technologies (IMRT vs. VMAT). No significant differences were found in the radiation dose between the two technologies for these ROIs. For patients treated with IMRT, the mean radiation doses were 55.84 ± 8.21 Gy, 53.53 ± 7.93 Gy, 55.65 ± 7.59 Gy, and 53.41 ± 7.26 Gy, respectively, in the left limbic lobe, left temporal lobe, right limbic lobe, and right temporal lobe. For patients treated with VMAT, the mean radiation doses were 55.93 ± 9.43 Gy, 54.03 ± 8.50 Gy, 55.04 ± 9.10 Gy, and 53.53 ± 8.53 Gy, respectively, in the same regions.

## 4. Discussion

In this study, we utilized the CT brain template developed by Christopher Rorden et al. (22) in the MNI space to investigate the impact of radiation on brain structure and function across the study population. Specifically, the CT brain scans and corresponding dose distribution maps of NPC patients were registered to this specific CT brain template, allowing for accurate identification of irradiated brain regions and evaluation of the dose distribution within these areas. Furthermore, we compared the dosimetry characteristics of specific normal brain tissues in NPC patients treated with different irradiation techniques, by analyzing group differences in RTdose images in the brain between the different treatment methods.

The preview of the projection of average RTdose maps into the MNI space showed that areas of the brain regions that received high doses (>50 Gy) of radiation were mainly located in parts of the temporal lobe (including a bit of both the superior and inferior temporal gyrus) and the limbic lobe (including the left and right uncus).

According to previous studies, brain regions that received high doses were prone to be damaged by the RT, resulting in RT-induced brain injury. Cheng-Yun Yao (36) et al. reviewed 327 patients with NPC receiving IMRT and found that 8 patients had radiation-induced brainstem injuries. Xiang Fan et al. (37) found that among 479 patients with NPC, six patients were diagnosed with RT-induced brainstem necrosis. They found the maximum dose (D_max_) of the brainstem in patients with RT-induced brainstem necrosis was greater than that in patients without necrosis. Further, Sheng-Fa Su et al. (38) found the incidence of temporal lobe injury is relatively high for patients with advanced T-stage NPC. Besides, the radiation could also induce subtle changes in the brain which could not be detected by the current clinical criteria. For NPC patients after RT, progressive diffusion was reduced in bilateral cingulate angular bundle fibers over time (39); significant and progressive radiotherapy-associated structural changes were detected in the bilateral temporal lobe (7); cortical thickness was also altered dynamically (40). These subtle changes may elucidate the pathogenesis of RT-induced cognitive decline. Most of these abnormal brain regions found in these studies had a tendency to receive higher radiation doses, which can be clearly shown on the dose distribution map in our study.

However, few of them discussed the direct relationship between specific structural or functional changes and the corresponding received dosage. Only one study revealed significant negative correlations between volume changes in the bilateral hippocampus, bilateral GCL, and right ML and the mean dose to the ipsilateral hippocampus (13). While in their study, the dosage information was extracted from the Dose-Volume Histogram (DVH) of the hippocampus, which was manually delineated on planning CT images. The volumes of structures were generated from ex vivo MRI data of postmortems. This method didn't make the structural information strictly correspond to its dosage information. Furthermore, the manual delineation of structures was very time-consuming, and the results of different physicians were inconsistent. By adopting the CT template created in MNI space, all patients' dose distribution maps could be registered into the same coordinate system, in which there were atlases for most structures in the brain. It will be more feasible to make the imaging information of structures strictly correspond to their dose information.

The analysis of volumes covered by VMAT and IMRT separately in different dose sub-ranges showed that the dose distribution pattern of IMRT and VMAT in the brain was complex and differed from the commonly held view that VMAT has a larger low-dose region than IMRT. However, these results were not strictly statistical and only represent a trend. We need to conduct rigorous statistical analysis to verify these results, which will be carried out in another study.

We believe that different radiotherapy techniques (IMRT or VMAT) result in different irradiation doses to brain structures, which inevitably leads to differences in invisible damage to brain structures. This is a direction for our future research. However, Tai-Lin Huang et al. found that these two treatment methods are consistent with tumor control, survival, and change of QoL for patients receiving IMRT and VMAT through follow-up visits. We suspect this result is because the EORTC QLQ-C30 and HN35 questionnaires used by Tai-Lin Huang et al. cannot reflect the functional changes of specific brain regions that received a certain dose of radiation very well (41).

The analysis for the dose of four ROIs (part of the left temporal lobe, the right temporal lobe, the left limbic lobe, and the right limbic lobe) indicated that (a) NPC patients treated with RT have high radiation dose in these four ROIs, including part of the superior temporal gyrus and the uncus, which are associated with uncal herniation syndrome and uncinate fits of mesiotemporal epilepsy; (b) in these ROIs the dosimetric performance of IMRT and VMAT techniques was similar. Associated with these regions, uncal herniation syndrome and uncinate fits of mesiotemporal epilepsy are the main neurological abnormalities (42). For example Yamei Tang et al. (43) found that Cystic lesions in the temporal lobes in MRI were more common in patients with epilepsy (18.74%). Radiation-related epilepsy may be caused by the high radiation dose in these four ROIs. The relationship of the radiation dose, the radiated regions and the radiation-related epilepsy occurrence could be explored more directly with our method.

The group analysis of RTdose data for different RTtechs of NPC patients showed significant differences in the RTdose distribution between IMRT and VMAT. The difference had the following characteristics: (a) for all patients, brain regions that received higher doses with IMRT were mainly distributed in the anterior region of the nasopharyngeal tumor (including regions near the temporal pole and orbital gyrus), while brain regions that received higher doses with VMAT were mainly located in the posterior region of the nasopharyngeal tumor (including areas near the cerebellum and occipital pole); (b) no significant difference was detected between T1 stage patients treated with IMRT and VMAT; (c) for T2 stage patients, brain regions that showed significant differences between IMRT and VMAT methods were widely distributed, and VMAT showed a significant dose advantage in protecting the normal brain tissue; (d) for T3 stage patients, brain regions that received higher doses with IMRT were mainly distributed in the superior temporal gyrus and the limbic lobe, while brain regions that received higher doses with VMAT were mainly located in the posterior cerebellum; (e) for T4 stage patients, VMAT showed a dose disadvantage in protecting the normal brain tissue. These results suggest that IMRT and VMAT have their own advantages in sparing different OARs in the brain for different T stages of NPC patients treated with RT.

Previous studies have reported inconsistent results. For instance, Szu-Huai Lu et al. (44) found that VMAT provided better sparing of the brainstem. Another study also found that the Dmax of the brainstem and temporal lobes was lower in VMAT (45). While Chen et al. found that the Dmax received by the brainstem of the VMAT was higher than IMRT. In this study, we found that the difference between VMAT and IMRT was complicated. VMAT did not show a definitive dosage advantage over IMRT in the brain. Our analysis objects were different from previous studies. By coregistering all patients' planning CTs and RTdose images simultaneously to a standard CT template, the dosage of OARs was compared by voxel, which was in the same location. In contrast, the Dmax of the OARs analyzed by previous studies was derived from a region. This difference may have led to diverse results. This inconsistency is noteworthy and needs to be further explored in future research.

However, several limitations should be noted in this current study. Firstly, we adopted a CT brain template created by Christopher Rorden et al. (22) in the MNI space, which was developed for the cohort with ages similar to what is commonly seen in stroke, based on 35 healthy old individuals. Significant differences have been found between Asian and Caucasian brain features (18, 46–48). Studies have shown that applying the template created from Western European or North American populations to Chinese individuals led to significantly greater deformation and reduced consistency compared with the use of a population-specific template (46–48). Furthermore, age, gender, ethnic, and sample size of the template also affected the segmentation and registration accuracy. The performance of the brain segmentation and registration would be significantly reduced when the mismatched templates were used (47–49). Therefore, a population-specific (e.g., age, ethnic, nationality) CT template should be created to promote the quality and accuracy of registration of structural and functional neuroimaging research for NPC patients in the future.

Moreover, all patients were from the same medical center. The result will be more universal by adopting multi-center patients’ CT and RTdose data. Thirdly, this study had not analyzed the relationship between cognition, dose, and imaging information, which should be explored carefully and deeply in the future.

## 5. Conclusion

Taken together, we present an approach for analyzing the dosimetric characteristics in a standard MNI space for Chinese NPC patients. We believed that this approach could provide a great convenience in the toxicity and dosimetry analysis for NPC patients with superiority localization accuracy.

## Data Availability

The raw data supporting the conclusions of this article will be made available by the authors, without undue reservation.
